# Prophylactic Cefazolin Dosing and Surgical Site Infections: Does the Dose Matter in Obese Patients?

**DOI:** 10.1007/s11695-018-3497-0

**Published:** 2018-09-29

**Authors:** Zahid Hussain, Colin Curtain, Corinne Mirkazemi, Karl Gadd, Gregory M. Peterson, Syed Tabish R. Zaidi

**Affiliations:** 1 Division of Pharmacy, College of Health and Medicine, University of Tasmania, Hobart, Tasmania Australia; 2 Department of Anaesthesia, Launceston General Hospital, Launceston, Tasmania Australia; 3 Head of Anaesthesia Discipline, Launceston Clinical School, University of Tasmania, Launceston, Tasmania Australia; 4 School of Healthcare, University of Leeds, Leeds, LS2 9JT UK

**Keywords:** Antibiotic prophylaxis, Cefazolin, Elective surgery, Obese, Surgical site infection

## Abstract

**Background:**

Most surgical prophylaxis guidelines recommend a 3-g cefazolin intravenous dose in patients weighing ≥ 120 kg. However, this recommendation is primarily based on pharmacokinetic studies rather than robust clinical evidence. This study aimed to compare the prevalence of surgical site infections (SSIs) in obese and non-obese patients (body mass index ≥ 30 kg/m^2^ and < 30 kg/m^2^), and those weighing ≥ 120 kg and < 120 kg, who received 2- g cefazolin preoperatively.

**Methods:**

A retrospective case-control study was conducted in adult elective surgical patients. Patients receiving 2- g cefazolin were grouped as obese and non-obese, and by weight (≥ 120 kg or < 120 kg). The 90-day prevalence of SSI and potential contributing factors were investigated.

**Results:**

We identified 152 obese (median 134 kg) and 152 non-obese control (median 73 kg) patients. Baseline characteristics were similar between groups, except for an increased prevalence in the obese group of diabetes (35.5% vs 13.2%; *p* < 0.001) and an American Society of Anaesthesiologists Score of 3 (61.8% vs 17.1%; *p* < 0.001). While not statistically significant, the prevalence of SSI in the obese group was almost double that in the non-obese group (8.6% vs 4.6%; *p* = 0.25) and in patients weighing ≥ 120 kg (*n* = 102) compared to those weighing < 120 kg (*n* = 202) (9.8% vs 5.0%; *p* = 0.17).

**Conclusion:**

The prevalence of SSI was not significantly increased in obese patients, or those weighing ≥ 120 kg, who received cefazolin 2- g prophylactically; however, trends toward an increase were evident. Large-scale randomised trials are needed to examine whether a 2-g or 3-g cefazolin is adequate to prevent SSI in obese (and ≥ 120 kg) individuals.

## Introduction

Obese patients undergo surgical procedures more frequently than their non-obese counterparts due to obesity-related health problems, such as osteoarthritis, cardiovascular disease, diabetes and cancer [1]. Obesity is also associated with a number of surgical complications, including an increased risk of surgical site infection (SSI) [2]. The repercussions of SSI include extended hospital stay, more frequent hospital readmissions, pain, anxiety and higher healthcare resource utilisation [3]. However, the administration of an appropriate antibiotic at an appropriate dose before surgery significantly reduces the risk of SSI [4].

Cefazolin remains the drug of choice for surgical prophylaxis in many procedures due to its favourable safety profile, low cost and targeted activity against the microorganisms commonly encountered during surgical procedures [4]. In 2013, a collective guideline for surgical prophylaxis developed by the Infectious Diseases Society of America (IDSA), American Society of Health-System Pharmacists (ASHP), Surgical Infection Society (SIS) and Society for Healthcare Epidemiology of America (SHEA), suggested an increased dose of cefazolin (3- g intravenously) for patients weighing ≥ 120 kg [4]. Similarly, the Australian Medicines Handbook (AMH) recommends a 3-g dose of cefazolin for patients > 120 kg [5]. The American Journal of Obstetricians and Gynaecologists (ACOG) Practice Bulletin and the Australian Therapeutic Guidelines (TG) also suggest the need for a higher prophylactic cefazolin dose for obese surgical patients but do not specify the recommended dose or weight or BMI cut-off values [6, 7].

The dosing recommendations of those guidelines were based on small-scale and inconsistent pharmacokinetic studies (level-III according to the National Health and Medical Research Council levels of evidence) [8]. Four pharmacokinetic studies found that a 2-g prophylactic dose of cefazolin may be inadequate in morbidly obese patients undergoing bariatric procedures and caesarean section, due to the blood and/or tissue drug concentrations being below minimum inhibitory concentrations (MIC) [9–12]. These studies suggested the need for a higher (3- g) dose in these patients. In contrast, six pharmacokinetic studies in similar surgical specialties found that a 2-g dose did provide adequate antimicrobial coverage (concentration above MIC) in morbidly obese patients with similar weight ranges, suggesting no dose increment was required [13–18].

Given the lack of satisfactory evidence supporting 3- g dosing in obese patients and a scarcity of clinical outcome studies, this study sought to ascertain whether an intravenous 2- g dose of cefazolin was comparatively effective in obese versus non-obese surgical patients, and in those who weighed above or below 120 kg, based on the observed rate of SSI within 90 days of operation.

## Method

A retrospective 1:1 case-control study was conducted of obese (BMI ≥ 30 kg/m^2^) and non-obese adults who underwent elective surgical procedures at the Royal Hobart Hospital (RHH) from 1 Jan 2012 to 31 Dec 2016. The prevalence of SSI at this institution was not known, so a duration-based (5-year) sampling method was used. The 500-bed RHH is the largest public teaching and referral hospital in the state of Tasmania, Australia. Ethical approval was obtained from the Tasmanian Health and Human Research Ethics Committee (H0015795). Informed consent from patients was not needed as data was collected retrospectively and de-identified upon collection.

Patients were included if they were at least 18 years of age and had received prophylactic cefazolin pre-operatively. The reasons for selecting elective cases were that more detailed documentation was available for these patients; they were more likely to have adequate pre-operative optimisation of medical comorbidities and a lower incidence of pre-operative bacterial colonisation compared to emergency cases [19, 20]. Patients were excluded if they (i) lacked follow-up within 90 days of surgery, (ii) had an unplanned non-infective post-operative intensive care unit admission, (iii) had a second operation during the same admission for causes other than infection, (iv) required perioperative blood transfusion, (v) were taking systemic immunosuppressive medication (corticosteroids, sirolimus, everolimus, cyclosporine, tacrolimus, azathioprine, mycophenolate, monoclonal antibodies or biologics, e.g. abatacept, etanercept) at admission and/or discharge, (vi) were receiving antibiotics immediately prior to admission or (vii) had missing requisite data (such as antibiotic type, dose, or surgical duration) in their medical record. Aside from BMI, the same inclusion and exclusion criteria were applied to obtain the non-obese (BMI < 30 kg/m^2^) control patients.

A list of obese patients, based on the International Classification of Disease-10 (ICD-10), who underwent elective surgical procedures during 2012 to 2016, was obtained from the hospital’s coding database. The list was then reviewed to identify patients who met the inclusion criteria. To include non-obese control patients, a list of similar elective surgical procedures from 2012 to 2016 was systematically screened, by including every fifth patient if they met the study inclusion criteria, until we reached approximately equal numbers in every surgical speciality to that of obese group.

Patients’ medical records were reviewed to obtain socio-demographic and clinical information, including gender, age, weight, height, body mass index (BMI), smoking status, diabetes status, length of stay (LOS), American Society of Anaesthesiologists (ASA) score [21], surgical wound class, duration of surgery, post-operative antibiotic use, surgical specialty and SSI incidence. Diabetes was identified based on a recorded diagnosis or use of any medication for diabetes management at admission or discharge. Wound class was categorised based on the Centre for Disease Control and Prevention Centre (CDC) criteria [22]. Duration of surgery was calculated as the time between skin incision and skin closure. LOS was calculated from date of admission until date of discharge in patients who did not develop SSI during admission or until date of SSI development for those who developed SSI during admission. Surgical procedures were grouped into a surgical specialty based on the department in which the patient underwent surgery, i.e. general surgery (such as laparoscopic adjustable gastric banding, laparoscopic cholecystectomy, incisional hernia repair), gynaecological surgery (such as caesarean section, hysterectomy, ovarian cystectomy) and orthopaedic surgery (such as hip and knee replacement, hip arthroplasty, ankle fracture). Prophylactic pre-operative cefazolin dose and post-operative antibiotic use (when not for SSI treatment) were recorded. Inpatient, outpatient and emergency department notes were screened for up to 90 days post-operatively to identify documented SSIs, which were classified into superficial, deep and organ/space, in accordance with the CDC [22].

Continuous variables were expressed as median (interquartile range) and categorical variables as the count (percentage). Pearson’s *χ*^2^ test and Fisher’s exact test were used for categorical variables, and the Mann-Whitney *U* test was used for continuous variables to compare the baseline variables and primary outcome. Logistic regression was used to identify the potential predictors of SSI. Variables (other than ASA score, post-operative antibiotic use and wound class) from the univariate analysis with a *p* value ≤ 0.20 were included in the multivariate logistic regression model. ASA score was not included in the multivariate analysis because it depends on two other study variables, diabetes and body weight. Post-operative antibiotic use was also not included in the multivariate analysis because its use was limited to certain surgical specialties, such as orthopaedics, and given to only 14% of patients. Wound class was also not included in the multivariate analysis because of the very small number of patients with contaminated and dirty wounds. The regression analysis was presented as unadjusted and adjusted odds ratios (OR) and 95% confidence intervals (CI). A *p* value of < 0.05 was considered significant in all the statistical analyses. Analyses were performed using SPSS version 22 (IBM Inc., Chicago, IL).

## Results

**Fig. 1:**
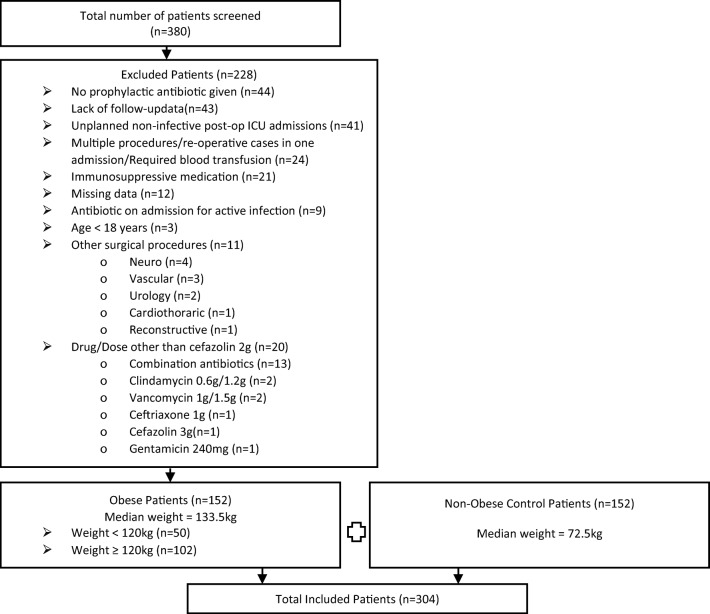
Flowchart of patient inclusion

One hundred and fifty-two obese patients met the inclusion criteria for this study and were matched with non-obese controls (Fig. 1). Patient characteristics are described in Table 1. There were differences between the obese and non-obese groups in median body weight (133.5 kg vs 72.5 kg, *p* < 0.001), median BMI (47.0 kg/m^2^ vs 26.7 kg/m^2^, *p* < 0.001), presence of diabetes (35.5% vs 13.2%, *p* < 0.001) and ASA score (score 3 in 61.8% vs 17.1%, *p* < 0.001). Overall, nearly two thirds (64.5%) were general surgical patients and more than half had a clean surgical wound in each group (non-obese = 58.6% and obese = 61.8%). Less than 2% of patients in either group were given prophylactic antibiotics when it was not recommended according to the Australian TG [7].

**Table 1:** Comparison of non-obese and obese patients

Variable	Non-obese (*n* = 152)	Obese (*n* = 152)	*p* value
Gender			
Female, *n* (%)	110 (72.4)	113 (74.3)	0.80
Male, *n* (%)	42 (27.6)	39 (25.7)	
Age (years), media*n* (IQR)	49.0 (30.0–61.0)	46.0 (31.2–54.0)	0.17
Weight (kg), median (IQR)	72.5 (65.0–82.0)	133.5 (115.0–148.0)	*< 0.001*
BMI (kg/m^2^), median (IQR)	26.7 (24.2–28.6)	47.0 (41.1–52.1)	*< 0.001*
Current smoker, *n* (%)	43 (28.3)	37 (24.3)	0.52
Diabetes, *n* (%)	20 (13.2)	54 (35.5)	*< 0.001*
ASA score			
1, *n* (%)	48 (31.6)	2 (1.3)	*< 0.001*
2, *n* (%)	78 (51.3)	56 (36.8)	
3, *n* (%)	26 (17.1)	94 (61.8)	
Length of stay (days), median (IQR)	2.0 (0.0–3.0)	1.0 (1.0–4.0)	0.14
Duration of surgery (min), median (IQR)	60.0 (45.2–89.5)	55.5 (45.0–90.0)	0.95
Implants, *n* (%)	78 (51.3)	77 (50.7)	1.000
Surgical specialty			
General, *n* (%)	98 (64.5)	98 (64.5)	1.000
Gynaecological, *n* (%)	41 (27.0)	41 (27.0)	
Orthopaedic, *n* (%)	13 (8.6)	13 (8.6)	
Wound class			
Clean, *n* (%)	89 (58.6)	94 (61.8)	0.91
Clean-contaminated, *n* (%)	61 (40.1)	56 (36.8)	
Contaminated, *n* (%)	1 (0.7)	1 (0.7)	
Dirty, *n* (%)	1 (0.7)	1 (0.7)	
Antibiotic prophylaxis recommended (as per TG [7])			
Yes, *n* (%)	149 (98.0)	150 (98.7)	0.65
Post-op antibiotic use			
None, *n* (%)	135 (88.8)	126 (82.9)	0.34
IV, *n* (%)	14 (9.2)	20 (13.2)	
Oral, *n* (%)	3 (2.0)	6 (3.9)	
Post-op antibiotic duration (h)			
IV, median (IQR)	20.0 (16.0–24.0)	24.0 (18.0–24.0)	0.36
Oral, median (IQR)	120.0 (48.0–160.0)	180.0 (120.0–240.0)	0.55

Statistically significant values (*p*<0.05) under the column of *p* values are shown in italics to highlight such significance

Thirteen (8.6%) obese and 7 (4.6%) non-obese patients developed SSIs (*p* = 0.25; Table 2). Similarly, the observed rate of SSI was 9.8% in patients weighing ≥ 120 kg (*n* = 102) compared to 5.0% in those weighing < 120 kg (*n* = 202) (*p* = 0.17). Three patients (2 obese and 1 non-obese) developed a SSI during their admissions and 17 (11 obese and 6 non-obese) developed SSI post-discharge. Four of the SSIs were classified as deep (2 obese and 2 non-obese) and 16 as superficial (11 obese and 5 non-obese).

**Table 2:** Relationship of SSI with patient and clinical characteristics

Variables	Prevalence of SSI (categorical variable) or median and IQR (numerical variable)	*p* value
Gender		1.00
Female, *n* (%)	15 (6.7)	
Male, *n* (%)	5 (6.2)	
Age (years), median (IQR)	SSI: 48.5 (28.7–54.0) No SSI: 47.0 (31.0–57.0)	0.86
Weight (kg), median (IQR)	SSI: 118.0 (74.5–139.2) No SSI: 94.5 (72.2–131.7)	0.18
BMI (kg/m^2^), median (IQR)	SSI: 40.7 (28.3–51.8) No SSI: 29.8 (26.5–46.9)	0.21
BMI category		0.25
Obese, *n* (%)	13 (8.6)	
Non-obese, *n* (%)	7 (4.6)	
Weight category		0.17
Weight < 120 kg, *n* (%)	10 (5.0)	
Weight ≥ 120 kg, *n* (%)	10 (9.8)	
Current smoker		0.90
Yes, *n* (%)	6 (7.5)	
No, *n* (%)	14 (6.3)	
Diabetes		0.06
Yes, *n* (%)	9 (12.0)	
No, *n* (%)	11 (4.8)	
ASA score		*0.02*
1, *n* (%)	1 (2.0)	
2, *n* (%)	5 (3.7)	
3, *n* (%)	14 (11.7)	
Length of stay (days), median (IQR)	SSI: 4.0 (1.2–5.7) No SSI: 1.0 (1.0–3.0)	*0.001*
Duration of surgery (min), median (IQR)	SSI: 80.0 (56.2–101.2) No SSI: 56.5 (45.0–89.5)	*0.02*
Implants		0.43
Yes, *n* (%)	8 (5.2)	
No, *n* (%)	12 (8.0)	
Surgical specialty		0.94
General, *n* (%)	13 (6.6)	
Gynaecological, *n* (%)	6 (7.3)	
Orthopaedic, *n* (%)	1 (3.8)	
Wound class		0.15
Clean, *n* (%)	10 (5.5)	
Clean-contaminated, *n* (%)	9 (7.7)	
Contaminated, *n* (%)	0 (0.0)	
Dirty, *n* (%)	1 (50.0)	
Post-op antibiotic use (other than for treating SSI)		0.067
No, *n* (%)	14 (5.4)	
IV, *n* (%)	5 (14.7)	
Oral, *n* (%)	1 (11.1)	
Post-op antibiotic duration (h)		
Oral, median (IQR)	SSI: 120.0 (84.0–240.0) No SSI: 120.0 (66.0–240.0)	0.90
IV, median (IQR)	SSI: 16.0 (16.0–48.0) No SSI: 24.0 (16.0–24.0)	0.64

Statistically significant values (*p*<0.05) under the column of *p* values are shown in italics to highlight such significance

Patients who developed a SSI had a significantly higher ASA score, longer duration of surgery and longer hospital LOS compared to patients who did not develop a SSI (Table 2). In the multivariate analysis, however, no variable showed a significant independent association with SSI (Table 3).

**Table 3:** Logistic regression for variables associated with SSI (*n* = 304)

Variable	Unadjusted OR (95% CI)	*p* value	Adjusted OR (95% CI)	*p* value
Weight category (weight ≥ 120 kg)	2.08 (0.83–5.19)	0.11	1.78 (0.69–4.59)	0.23
Diabetes	2.70 (1.07–6.80)	0.04	2.31 (0.88–6.06)	0.09
Length of stay	1.18 (1.03–1.35)	0.01	1.13 (0.97–1.33)	0.11
Duration of surgery	1.00 (0.99–1.01)	0.09	1.00 (0.99–1.01)	0.43

## Discussion

The dose of prophylactic antibiotic is an important factor in SSI prevention, and pharmacokinetic studies provide baseline information about dose and timing. However, pharmacokinetic findings may not always be translated into clinical outcomes [23]. Our findings showed no statistically significant difference in SSI prevalence between obese and non-obese patients, or those who weighed above and below 120 kg, who received a 2-g prophylactic cefazolin dose preoperatively. However, there were approximately twofold increases in SSI prevalence in obese compared to non-obese patients and in those who weighed ≥ 120 kg compared to those who weighed < 120 kg. The lack of statistically significant differences could be due to our relatively small sample size.

To date, no outcome study has shown the superiority of using a dose of prophylactic cefazolin exceeding 2- g in obese surgical patients. A retrospective outcome study was conducted of obese (mean BMI = 35 kg/m^2^; *n* = 99) and non-obese (mean BMI = 27 kg/m^2^; *n* = 96) patients across various surgical specialties, who received a 2-g cefazolin prophylactic dose [24]. No significant difference in 30-day SSI prevalence was noted between the obese and non-obese groups (7.0% vs 5.2%, *p* = 0.56) [24]. Likewise, a recent retrospective study of 2- g (mean BMI = 36 kg/m^2^; *n* = 152) or 3- g (mean BMI = 40 kg/m^2^; *n* = 284) prophylactic cefazolin dosing in obese patients of various surgical specialties reported a very similar 90-day SSI prevalence in the two dosing groups (7.2% vs 7.4%, *p* = 0.95) [23]. Our obese cohort had a higher median BMI (47 kg/m^2^) compared to patients in the aforementioned studies (35 kg/m^2^ and 36 kg/m^2^) who received a 2-g cefazolin dose. This is a possible explanation for the larger difference in SSI prevalence in the obese patients versus control patients in our study compared to the previous studies [23, 24].

Appropriate prophylactic antibiotic administration is just one measure of the multifactorial approach used in the prevention of SSI. Therefore, stringent inclusion criteria on patient selection were applied so that the effect of cefazolin dosing could be independently estimated. We excluded patients with factors that can potentially alter the pharmacokinetic properties of antibiotics (such as non-infective unplanned post-operative admissions due to acute illness [25]) or affect the wound healing process (such as peri-operative blood transfusion and taking immunosuppressive medications [26]). Furthermore, non-modifiable risk factors, such as older age, smoking, diabetes, LOS, duration of surgery, pre-existing implanted medical devices and wound class, were considered in the statistical analyses.

Patients with diabetes, an ASA score of 3, longer surgery duration and longer LOS tended to have higher SSI occurrence in our study. These are well-established known risk factors for SSI development [27]. Other SSI risk factors reported in the literature, such as smoking, advanced age and non-clean surgical wounds [27] in obese patients, did not show a significant association with SSI.

This study has some limitations. Firstly, it was not a prospective randomised controlled trial. The retrospective study design meant we had to rely on the notes available in patients’ medical records. For instance, we were not able to record the exact timing of prophylactic cefazolin dose administration. However, from the anaesthetic chart reviews, we could ascertain that the doses were always administered in theatre, anywhere from immediately before induction until shortly after incision. As mentioned, the sample size of the study was relatively small. One possible reason for the small number of patients identified in our case group is that obesity was coded sporadically in hospital records as a comorbidity (ICD-10 list). Also, we excluded patients who underwent vascular, urologic, cardiothoracic and reconstructive surgery due to their limited numbers, which might compromise the generalisability of findings to these surgical specialities.

## Conclusion

While no statistically significant difference in SSI prevalence was observed in non-obese and obese patients, or those who weighed above and below 120 kg, who received a 2-g prophylactic cefazolin dose, trends toward an increase were evident. There is a clear need for large-scale randomised controlled trials to examine whether a 2-g or 3-g cefazolin dose is adequate to prevent SSI in obese individuals.
